# Can Rural Migrant Workers Afford to Retire in China? A Study of Chinese Migrant Worker’s Retirement Savings

**DOI:** 10.1093/geroni/igab046.1753

**Published:** 2021-12-17

**Authors:** Jing Liu, Heying Zhan, Fengxian Qiu

**Affiliations:** 1 Zhejiang University of Finance and Economics, Hangzhou, Zhejiang, China (People's Republic); 2 Georgia State University, Georgia State University, Georgia, United States; 3 Anhui Normal University, Wuhu, Anhui, China (People's Republic)

## Abstract

This paper makes connections between social policies of retirement, migrant worker’s migration experience, and migrant workers’ retirement savings. Using insight from the political economy of aging and stress theory, this paper links the macro levels of understanding with the micro levels of work and aging experiences for migrant workers. Using binary logistic regression with a sample of 699 Chinese migrant workers from three emigration provinces (Anhui, Henan, Sichuan), this paper explores four specific aspects of migrant worker’s migration experience in relation to their retirement savings: financial status; length of employment; social support, and levels of hopefulness. Findings reveal that migrant workers with better financial status, social support, and higher level of hopefulness towards future are more likely to have retirement savings as compared to their counterparts. Discussions linking the macro and micro levels of social policies were provided. Policy implications were discussed.

